# Informal settlements and the care of children 0–3 years of age: a qualitative study

**DOI:** 10.3389/fpubh.2023.1110578

**Published:** 2023-08-23

**Authors:** Thivia Jegathesan, Aisha Yousafzai, Michaela Mantel, Vittorio Sereni, Robert W. Armstrong, Ripudaman Singh Minhas

**Affiliations:** ^1^Department of Pediatrics, St. Michael's Hospital, Unity Health Toronto, Toronto, ON, Canada; ^2^Department of Global Health and Population, Harvard T.H. Chan School of Public Health Department, Boston, MA, United States; ^3^The Health Associates GmbH, Berlin, Germany; ^4^Janssen-Cilag SpA, Milan, Italy; ^5^Department of Pediatrics, University of British Columbia, Vancouver, BC, Canada; ^6^Aga Khan University, Nairobi, Kenya; ^7^Temerty Faculty of Medicine, Division of Developmental Pediatrics, Department of Paediatrics, University of Toronto, Toronto, ON, Canada; ^8^Li Ka Shing Knowledge Institute, Unity Health Toronto, Toronto, ON, Canada

**Keywords:** babycare, child care, early child care, resource poor environments, informal urban settlements, Kenya, low- and middle income country

## Abstract

**Background:**

There is a rapid increase in urbanization with a high percentage of people living in poverty in urban informal settlements. These families, including single parents, are requiring accessible and affordable childcare. In Mlolongo, an informal settlement in Machakos County in Nairobi metropolitan area, Kenya, childcare centres, referred to as ‘babycares’ are increasing in number. They are being provided by local community members without attention to standards or quality control. The study objective was to understand parents’, caregivers’ and community elders’ experiences and perceptions in terms of the quality of babycares in Mlolongo to inform the design and implementation of improved early childcare services.

**Methods:**

Using a community-based participatory research philosophy, a qualitative study including focus group discussions with parents, community elders and babycare centre employees/owners (referred to as caregivers) was conducted in Mlolongo.

**Results:**

A total of 13 caregivers, 13 parents of children attending babycares, and eight community elders participated in the focus groups. Overall, community elders, parents and caregivers felt that the babycares were not providing an appropriate quality of childcare. The reported issues included lack of training and resources for caregivers, miscommunication between parents and caregivers on expectations and inappropriate child to caregiver ratio.

**Conclusion:**

The deficiencies identified by respondents indicate a need for improved quality of affordable childcare to support early child development in these settings. Efforts need to be invested in defining effective models of early childcare that can meet the expectations and needs of parents and caregivers and address the major challenges in childcare quality identified in this study.

## Introduction

The environment in which a child grows, plays, and receives care is vital to fostering its developmental potential (1). With rapid urbanization occurring globally, more working families are requiring accessible, affordable childcare (2). Parents in poor urban informal settlements are particularly challenged given their separation from the extended rural family and the severe financial constraints, the lack of trained childcare caretakers, poor infrastructure and hygiene situation, and security issues in these areas. Innovative intervention strategies that support safe, nurturing and quality childcare in these low-resource settings are necessary to enable parents to go out of their homes to work and provide for their family while simultaneously being sure that their children are well taken care of and receive a sound foundation for their development.

The United Nations Sustainable Development Goal 4.2 aims to ensure that all children have “access to quality early childhood development, care and pre-primary education, in preparation for primary education by 2030,” (3). The aim is to ensure all children around the world have equal access to affordable childcare. The implementation and expansion of childcare programs has been reported as insufficient to reach this goal, both in the absolute number and the quality of childcare centres. To achieve this goal, an equity-based approach to achieve universal childcare should be applied, where the quality of childcare is measured from an ecological perspective (4). Quality of childcare programs can be achieved through the design, curriculum, training for childcare workers, monitoring and assessment of programs, and appropriate governance and supervision (5).

### Kenya and the state of childcare in urban informal settlements

Kenya has been ranked as a lower middle-income country since 2014 (6) with a rapid urbanization reaching 28.5% in 2021 (7). Approximately 22% of the residents in Kenya’s capital city Nairobi live in severe poverty with 60–70% of city dwellers (about 2.5 million) living in some 200 informal settlements (8). Most families and individuals in these resource-poor communities lack access to essential services such as water and sanitation, health services, quality education, social services, and economic empowerment.

With an increasing majority of Africa’s populations living in urban settings, financial constraints, poor housing conditions, and lack of infrastructure can all significantly impact young children’s long-term development. The challenge is finding workable models to provide affordable and developmentally supportive care for these children.

There are a number of different childcare options in Kenya, ranging from formal care (e.g., private nurseries and child care centres) to informal care (e.g., siblings, grandparents and other family members) (9). UNESCO stated “the care and education of young children under three in Kenya is largely in the hands of older siblings, grandparents and house help, if they are available” (10). However, traditional extended family members may not be available to support childcare especially for young families migrating from their rural homes to cities like Nairobi. At the time of the study many mothers lacked maternity leave benefits, or at best, received only the government mandated 12 weeks (11, 12), and therefore needed appropriate childcare options when they returned to work.

For children over 3 years of age, the Kenyan government introduced free pre-primary education at developmental care centres. However, the “Education Sector Strategic Plan and Implementation Matrices” (Kenya, 2003–2007) was not implemented as planned resulting in the private sector taking over childcare provision (9). Most recently, there has been a shift to group childcare in the form of “care centres” due to the high costs and perceived problems with nannies in private homes (9). There is little known about the quality of these centres and there is limited knowledge about the number of childcare workers in Kenya and their standards of practice (13). Studies that exist have revealed poor hygiene, poor feeding, and varying degrees of quality across sites (14). The lack of standards in childcare centres and large inconsistency in resources and staff training is problematic. This puts enrolled children at risk of compromised developmental potential due to poor quality of care and safety standards.

Nairobi’s informal settlements have continued to brim with economic migrant families, often necessitating that mothers and older children seek employment outside their homes, leaving younger children in need of appropriate childcare options. In these areas, single mothers experience ‘elevated stress’ with little financial support by the fathers and the comparatively weak social support available in these precarious urban environments (15). A study in an urban informal settlement in Nairobi showed that “mothers employ three main strategies to balance their work and child care responsibilities: (1) combine work and childcare, (2) rely on kin and neighbors, or (3) use centre-based care” (16).

### Mlolongo County and early childcare

Mlolongo, is a city in Machakos County, and part of the Nairobi metropolitan area situated about 14 kilometers from Nairobi. The Nairobi to Mombasa highway traversing the Mlolongo mid-stream is the commonest feature here. At the time of the study the population of Mlolongo was estimated to be 100,000 (17) and children 0–4 years of age represented 7.8% of the Kenyan population (18). It is a densely populated area with more than 40 Kenyan tribes represented, including other nationals from East Africa and beyond. Being part of the sub-urban areas around Nairobi, the languages spoken here are national. Most residents are day laborers, small business owners, employees of local businesses, cleaners, and housemaids in households in richer areas and street vendors.

In the informal settlement of Mlolongo, specific information about childcare is scarce. The Orphans and Vulnerable Children (OVC) project was the first one of its kind in Mlolongo supporting childcare until 2012. The OVC staff carried out some informal unpublished assessments of the so called private “babycares” in the area (17). The assessments revealed that centre-based childcare was available in a variety of options ranging from childcare centres organized by non-government organizations to unofficial ‘babycares’ run by informal settlement dwellers and local proprietors themselves. Most of centre-based care was fee-based and larger centres with trained staff were often not affordable for the parents in these areas.

The babycares industry in Mlolongo was relatively new and grew quickly to meet the demands of the burgeoning population, with little or no oversight, training, or support. Babycares were independently established by individual community members and proprietors with no training in child development or in entrepreneurship. At the time of the study, Mlolongo had 70 private small enterprise babycares taking care of approximately 1,400 children of age 3–36 months (19). An average of 22 children were found in a babycare with an average of 15 children per caregiver. Parents (often single mothers) would drop off their young children in the early morning prior to leaving for their own work and pick them up in the evening. Depending on their incomes, they paid fees to the caregivers but often failed to do so. The OVC assessments of the babycares identified profound inadequacy including lack of developmental programming; poor nutrition for the children that had to be provided by the parents; poor hygiene conditions; lack of space; little or no training of caregivers in childcare and thus huge skills gaps in caretaking of the children’s basic needs.

In 2012, an informal visit by the researchers to Mlolongo babycares before the start of this research project revealed extremely unconducive environments and unbearable conditions for the young children taken care of in mostly private babycares (20). Furthermore, the assessment showed poor community understanding of the importance of basic quality in early childcare and lack of childcare standards or regulation with significant variability in practices and resources to support nutrition, development, and play. Financial challenges were observed leading to overcrowding and poor environments, and children were abruptly displaced with unexpected closure of babycares if the owner attained more lucrative employment. However, for a lot of working mothers, the options were either these make-shift daycares or leaving their infant unsafe and unguarded at home alone or with an older sibling who had been pulled out of school.

A follow-up study of 30 mothers with six to36-months-old children and using Mlolongo babycares revealed that the economic condition of families was dependent on casual labor but was better than in other urban settlements in terms of household stability (two parents), number of children per household and monthly income (19). The study showed that 73% of mothers were married and that there were an average 1.7 children per household and 14,300 Kenya Shillings (KES) average family income. Only 17% of mothers and 33% of fathers had a permanent job. There was a significant economic impact (family lost income) related to child illness, with 4.8 days/month spent with sick children and 76% of mothers who did not go to work if the child was sick, amounting to an average daily income loss of 392 KES.

## Aim of the study

This study carried out in 2013 was the first of a series of studies under an implementation research project carried out between 2013 and 2015 to improve childcare for infants and young children in Mlolongo, an informal urban settlement at the outskirts of Kenya’s capital city Nairobi. The objective of the study was to understand parents’, caregivers’, and community elders’ perceptions of and experiences with the current quality of babycares in Mlolongo, ensuring that the community is engaged and has a voice in the design to achieve quality affordable and sustainable childcare in their community.

## Methods

### Design

The study team used a qualitative phenomenological design to obtain an understanding of key stakeholders’ perspectives and experiences of babycare centres quality with the intent to co-create solutions to this problem in subsequent phases of the project (22). Parents (consumers), caregivers (providers) and community elders (public beneficiaries) were involved in the design of the interview guide for focus group discussions, participated in focus group discussions (FGDs) and provided feedback on gaps in the current system. Using this approach, the study sought to explore the current knowledge, behaviors and practices pertaining to childcare, and explore existing challenges, through lived experiences of the stakeholders. In this study, a caregiver is defined as an owner or worker of babycares who provides childcare services for parents of infants and young children. Participating parents were defined as community members using the babycares for the care of their children when at work or otherwise busy.

### Participant selection and setting

Using purposive sampling, eligible participants for the FGDs were identified by research staff and caregivers and contacted in person at the babycares located within the informal settlements of Mlolongo, Kenya. Prior to recording the FGDs, the moderator explained the details of the focus group content, noted that the discussion would be recorded and transcribed and assured participants that transcription of participant comments would be anonymized. Focus group composition was homogenous, and conducted separately with each stakeholder group (caregivers, parents, and community elders) to maximize the differing perspectives each community group might bring to the understanding of babycares.

### Data collection

Aligned to the WHO standards for caregiving, moderators explored the quality of the babycares through a semi-structured interview guide that sought to cover the following topics: (1) current process and operations of babycares, and (2) caregivers’ and parents’ perception of their roles and responsibilities, the moderator asked questions related to these topics and probed additional questions to get a better understanding of participants’ lived experiences working and participating in babycares. The interview guide is presented in Appendix A.

### Focus group details

Focus groups were conducted in Kiswahili at a central location in the Mlolongo community for 1 h each, with one moderator and one recorder. The moderator was responsible for engaging all participants encouraging them to share their experiences working or using babycares. Moderators obtained informed consent from participants and ensured participants were aware that participation was voluntary. The FGDs were audio-recorded, and moderators took field notes during the discussion.

### Data management

Transcript recordings were kept on a locked server at the Aga Khan University in Nairobi, and transcriptions were de-identified. During the transcription of focus groups, participants’ names and identifying information were not recorded. They were identified only by gender (male or female) and stakeholder (e.g., caregiver, parent, community elder/leader). FGD audio-recordings were transcribed in Kiswahili and translated to English by an experienced interpreter/translator. This was verified by an additional researcher for accuracy.

### Analysis

The Consolidated Criteria for Reporting Qualitative (COREQ) research guidelines were followed in reporting the qualitative enquiry (21). An iterative thematic analysis approach was adopted following guidelines from Creswell & Poth (22). All participants took part in one-time FGDs with no long-term commitments for subsequent project involvement. Two analysts first reviewed transcripts from interviews with caregivers, parents, and community elders. A continuous review of interview data informed whether a point of saturation was reached (i.e., no new information was yielded) whereby after each, FGD was completed and transcribed, transcripts were reviewed, and saturation was determined to be reached when themes began to consistently overlap. Using a process of memoing, patterns in the data were identified and initial codes were created inductively. The codes were discussed and classified into themes based on the discussions by the analysis about what was learned about the lived experiences of interviewees about the babycares, aligning with the phenomenological design of the study. Additionally, themes were also identified deductively using codes informed by the WHO standards for child care service (22). The analysts then independently coded the remaining transcripts and reviewed together. Linkages and groupings among themes were discussed to create the final set of themes.

### Research team and reflexivity

The research team was made up of individuals from a diverse background of expertise who participated as the moderator in focus groups and analyzed the transcripts. The moderator was a local Kenyan student. The analysis team included a developmental pediatrician, a child health scientist, a graduate student, local senior program advisor in Kenya and professor of pediatrics at Aga Khan University.

## Results

### Participants

A total of five FGDs were conducted, consisting of six to eight participants per session. The FGDs included: 13 caregivers of babycares (two FGDs), 13 parents (two FGDs) of children attending babycares, and one group of eight community elders. All caregivers and parents were female, whereas all community elders were male.

### Characteristics of caregivers and babycares

To protect the privacy of the participants the names of the centres will not be revealed and only the types of sites will be mentioned in this paper. Caregivers worked at schools and as community health workers. They took care of between 8–18 babies and children at one time. One caregiver took care of fewer than eight babies and children, while the remaining seven took care of greater than 10 babies and/or children. The children they cared for were between 6 months and 3 years old, with workdays beginning between 6 am and 7 am, and ending between 7 pm and 8 pm. Their hours depended on how long each parent left their individual child at the centre. Many of these babycares were reportedly conducted in the caregiver’s home and caregivers completed several duties and tasks during this time, which included, but were not limited to (1) feeding the baby, (2) bathing the baby, (3) putting the baby to sleep and (4) some play with the babies. However, the nature of work and the number of babies caregivers cared for in the centres varied.

In response to the following question “How many babies do you have at the babycare,” (Moderator, FDG 1), the following responses were provided:

*“I have around 13 babies ranging between 6 months to 3 years*. *I have 2 caregivers*.*”* (*Caregiver 1, FDG2*).

*“I have around 18 babies ranging between 4 months and two and a half years*. *I have 2 caregivers*.*”* (*Caregiver 2, FDG2*).

*“30 and [I] am alone,”* (*Caregiver 2, FDG1*).

### Characteristics and responsibilities of parents

Thirteen parents (only mothers) participated in two separate focus groups as clients of Mlolongo’s babycares. Mothers were all from the same community of Mlolongo. Nine out of 13 of these mothers were working. Two of these mothers worked as caregivers at other babycares, and one mother worked at a boutique. The occupation of the remaining six mothers was not mentioned.

Mothers’ roles and responsibilities differed between babycares, but typically parents were expected by the caregiver to bring enough food for the baby throughout the day, and other personal items of the baby. Most of the mothers fed their babies in the morning and dropped them off at babycares between 7 am and 9 am in the morning. Most mothers did not report a specified time at which they picked up children and mothers indicated that there was usually a set fee for the day; one mentioned she gets the baby between 6 pm and 7 pm. Most mothers prepared food for their babies, but, identified that, if there is not enough food for the day, the caregiver would provide food for the baby. Overall, parents expected caregivers to do the following at babycares: (1) cook and provide food for the baby, (2) play games, (3) put them to sleep, (4) change their diapers and bathe them, (5) administer any medication the baby may require, and (6) fulfill all other needs the baby may have during his/her stay. The cost of babycares was dependent on the caregivers’ individual duties and requirements. However, there was no standard pricing for these tasks. Some caregivers would do “additional” work like start earlier than they are scheduled to accommodate parents that attend the babycares early. When caregivers were asked about the tasks they complete and their schedules, their responses included the following:

*“I begin baby care by 6*.*00 am because I do it in my residential house*. *I clean it well, spread their bed, at 9*.*00 am I give them milk, change them, put them to sleep, wake them up, change them, feed them, we go out to play, I change them then their parents start picking them from 4*.*00 pm*. *The last baby is picked at 8*.*00 pm*. *You cannot charge some parents more even if they pick their babies* [*late*] *because they are not financially able to pay more,”* (*Caregiver 1, FDG2*).

*“My baby care starts at 6*.*00 am but some babies are brought at 5*.*30 am…*.*,”* (*Caregiver 2, FDG2*).

### Perception of community leaders

Eight community leaders participated in one focus group discussion. Each elder came from eight different villages in Mlolongo. The villages are not disclosed in this paper to protect the privacy of the community leaders. All community leaders endorsed that babycares were currently functioning at low quality with limited resources. Community leaders felt caregivers needed more training, support, and resources to ensure the safety of the babies who attend the babycares. They expressed their concerns around the differing roles each caregiver plays, making it difficult to understand what caregivers do. They felt parents had varying reasons for using babycares. These included perceptions that parents had low incomes and could not take time off work to care for their child. In addition, babycares were perceived to be a more reliable form of childcare than “house girls,” who were usually maids/nannies within the parent’s home.

Community Leaders perception of babycares:

*“The standard of the baby care is very low so I would opt that food is brought for them”* (*Elder 2, FDG1*).

*“Baby care should not be in a small room*. *A place should be set aside for them like in a church where there is enough space,* “(*Elder 1, FDG1*).

Community Leader perceptions of why parents use babycares:

*“The salary these parents earn is very low*. *Their income is minimal so they are not in a position to hire a domestic worker so they take them to baby cares that they can afford to pay*.*”* (*Elder 1, FDG1*).

*“Domestic workers are untrustworthy so some parents fear for example they* [*cannot*] *leave the baby alone in the house and go away*.*”* (*Elder 3, FDG1*).

### Themes

Three major themes emerged around the quality of babycares in Mlolongo informal settlement. See Table 1 for a summary of major themes and subthemes identified in this study.

**Table 1:** Major themes and subthemes identified on quality of babycares in Mlolongo, Kenya.

**Major Themes**	**Subthemes**
Babycares play a role in addressing issues experienced by parents when providing childcare	Maternal low-income and nature of employment prevents the ability to take time off from work. Nature of employment and income of mothers contribute to the mother’s selection of babycares. Elders and caregivers cite maternal stress, immaturity and nature of parenting as reasons for seeking babycares.
Current quality of babycares and improvements required	Inappropriate babies to caregiver ratio. Limited space and lack of cleanliness of babycares. Caregivers not provided with appropriate and continuous training.
Systemic barriers to resolving poor quality and establishing standards	Disconnect and disagreement between parents, caregivers and elders.

**Theme 1**: Babycares play a role in addressing issues experienced by parents when providing childcare.

- There is tremendous need for babycares in the Mlolongo community.

Mothers undergo an immense amount of stress and pressure after their child is born, given the regulated maternity leave available. At the time of the study mothers were limited to 12 weeks of maternity leave. Babycares are sought to help with issues experienced by parents when raising their child related to the socioeconomic status and personal characteristics of the parent. The factors resulting in the need for babycares were reported from the perception of parents, caregivers, and community leaders. While parents focused more on their nature of employment and income, caregivers and community leaders attributed use of babycares to the mother’s employment and maternal stress. In addition, babycares were seen as highly accessible due to proximity to clients’ homes or workplaces.

- Maternal low-income and nature of employment prevents the ability to take time off from work.

Many mothers in this study who were engaged in low-income employment were unable to take time off to care for their baby, and, as a result, sought affordable childcare options. Some parents owned shops or worked in a private business requiring them to be there for more than 8 h a day.

*“I sell in a boutique*. *I get there at 8:00 am and leave at 7:00 pm,”* (*Parent, FG1*).

Parents feel babycares are trustworthy and less expensive than hiring a single babysitter or caregiver to watch their child at their house. These individuals are called “house girls,” who come over to the parents’ house and watch the child while the parent is away. Parents report that this option has now become expensive and, at times, unreliable for parents. One parent said:

*“I would say babycare is the best, since nowadays hiring a house girl is such an expense*. *For example, I had a house girl who burnt my baby, so staying at the hospital with the baby was expensive*.*”* (*Parent, FG1*).

The elders also agree with the parents’ perception of babycares being more affordable than other childcare centres.

*“The salary these parents earn is very low*. *Their income is minimal so they are not able to hire a domestic worker so they take them to babycares that they can afford to pay*.*”* (*Elder, FG1*).

Elders in the community agree with parents and babycare centre caregivers on the benefits of babycares in helping parents who may not be able to be with their baby due to work.

*“The parent can easily fend for the baby because she has a person, she can leave her baby with*. *The baby gets mother’s love even in the absence of their real mother,”* (*Elder, FG1*).

- Nature of employment and income of mothers contribute to a mother’s selection of babycares.

Parents reported several considerations before they opted to use babycare centres. These considerations also determined the type of babycares they chose, suggesting most parents have their own set of standards they expect babycares to meet. Four out of five mothers reported the primary reason for taking their child to a babycares was because they were currently employed at jobs that took up most of their day, typically from 8:00 am – 6:00 pm. They all highlighted that being away from their baby during the day required securing external help to care for their child. While some mothers felt that babycare centres were a less expensive option for providing care for their child while they were away, other mothers suggested that hiring a house girl was a potential alternative for flexibility. Some mothers reported the benefits to hiring a house girl, being able to stay longer at work and reduced amount of stress getting their child ready to drop off at babycares. However, mothers did indicate that they were more drawn to babycares because they were more affordable. Mothers reported if they opted to take their child to babycares over hiring a house girl, they expected the babycares to meet a number of criteria.

One parent said the following when choosing a babycare centre:

*“Some of us, we consider cleanliness*. *You compare different babycares and in case you discover one does not change nappies, which can cause nappy rash, you be very careful*. *A congested place is also not conducive for the baby*.*”* (*Parent, FG2*).

When asked about how much the cost of a babycare centre influences their decision one mother said.

*“There are instances where it does, and it does not*. *The caregiver can charge fifty shillings and she is clean, while another one charges 100 shillings, but is not as clean as the cheaper one*.*”* (*Parent, FG2*).

This highlights the impact of financial and social situations on expectations and decisions regarding babycares.

The elders felt that the mothers’ educational background, and profession may impact their decision to use babycares.

*“The nature of work the parents do, for example, others are commercial sex workers, bar maids so instead of locking up the babies they take them to babycare*.*”* (*Elder, FG1*).

Elders and caregivers cite maternal stress, immaturity, and nature of parenting as reasons for seeking babycares.

Caregivers and elders have negative opinions of parents seeking babycares. They feel parents who seek babycares do not want to take responsibility for their child or are disadvantaged and as such are required to work instead of being able to look after their child.

*“*[*We see*] *irresponsible parenting like drunkenness, so there is no attachment and others are very poor, so busy looking for money, so they just leave the baby at babycare*.*”* (*Caregiver, FG1*).

However, they do realize that babycares are more trustworthy than traditional use of house girls.

*“Caregivers are also more mature than these girls, so some people are preferring them,”* (*Caregiver, FG1*).

**Theme 2:** Current standards and quality of babycares and required improvements.

Caregivers, parents and elder’s opinions were sought to understand their perceived thoughts about the quality of babycares. These included: (1) inappropriate baby to caregiver ratio, (2) limited space and cleanliness of babycares, (3) lack of appropriate training provided to caregivers, and (4) expected standards and quality of babycare.

- Inappropriate babies to caregiver ratio.

Parents, caregivers working at the babycares and elders highlighted the inappropriate baby to caregiver ratio.

“*You find one person taking care of about 15 and 20 babies*. *We need like 1:5*. *The small ones need a lot of attention due to crying and changing*.*”* (*Caregiver, FG1*).

*“Babies should be separated in accordance to their ages and cared for by different people not one person for all of them*.*”* (*Elder, FG1*).

- Limited space and lack of cleanliness of babycares.

Parents and elders reported babycares to be limited in space and unclean. Babies have also been reported to sleep in uncomfortable environments including on desks and seats. They have reported babies to be susceptible to communicable diseases by sharing spoons and plates.

*“There is a risk at babycares because there is no checking on the health status of the baby*. *Besides HIV, there are also communicable diseases, and these can be very contagious*. *Measures should be taken to address such issues*.*”* (*Caregiver, FG1*).

*“Sharing spoons and plates should be avoided to curb spread of contagious diseases*.*”* (*Parent, FG1*).

*“The problem I see is that the room is tiny and there are more than 10 children*. *The babies sleep on the cold floor; hence they do not sleep well since there are no sheets or blankets*.*”* (*Elder, FG1*).

Caregivers have reported similar concerns about the setting in which they are required to provide care to these babies. In addition to the inappropriate baby to caregiver ratio, they also raise the problem of long hours with workdays ranging in length between 8 and 12 h with few resources, training, and support.

One caregiver described her day stating.

*“I begin babycare by 6:00 am because I do it in my residential house*. *I clean it well, spread their bed, at 9:00 am I give them milk, change them, put them to sleep, wake them up, change them, feed them, we go out to play, I change them, then their parents start picking them from 4:00 pm*. *The last baby is picked at 8:00 pm*. *You cannot charge some parents more even if they pick their babies late because they are not financially able to pay more*. *They play with dolls and run after each other*. *Those very young ones just eat and sleep; they do not play,”* (*Caregiver, FG1*).

- Caregivers not provided with appropriate and continuous training.

Parents and elders felt some caregivers were not trained to take care of their babies. Furthermore, some expect the caregivers to administer medicine, which they feel they are not adequately trained to do.

*“Caregivers need to be trained on caring for babies*. *and have laid out standards and should have a license*. *Babies should have a medical record and in case of sickness there should be first aid or a clinic nearby where they can receive treatment*.*”* (*Elder, FG1*).

*“The babies’ temperatures rise frequently hence every other day you take the baby to hospital and are put on medication*. *There is no medicine for emergency, so the caregiver waits until evening, you take your baby to hospital…She does not know how to give medicine*.*”* (*Parent, FG2*).

Caregivers reported having varied educational and training backgrounds. This included: (1) Sunday school, (2) mentoring, (3) seminars, (4) nursing, and (5) Orphaned and Vulnerable Children (OVC) training. The OVC training program was a previously funded program organized by OVC staff and a private donor that were funded by small donations from charities in the US and Mlolongo (23). Caregivers expressed interest in obtaining additional formal training in early child development. They reported learning from each other by visiting each other and sharing ideas and concerns about their practice.

When asked what training caregivers preferred, caregivers listed:

*“Professional presentation, ECDE and Child Development”* (*All caregivers, FDG2*).

*“Anything new*. *Knowledge is power*.*”* (*Caregiver 1, FDG1*).

One caregiver also stated they visit each other at babycares and learn from each other.

*“Yes*. *When we visit each other we enlighten each other and help each other mentally*.*”* (*Caregiver 1, FDG2*).

- Expected standards and quality of babycares.

Parents and elders felt that babycares should abide by set standards surrounding hygiene, resources and training, such as those outlined:

Parent and Elder Perspectives on Babycare Standards.

- Always ensure cleanliness.
- Diapers should be provided by caregivers.
- Food should be prepared on site in babycares.
- Toys to play with for child should be available.
- Trained caregivers should be hired with completed background checks.
- A consistent schedule should be provided of when babycare hours commence and end.

**Theme 3**: Systemic barriers to resolving poor quality of babycares.

When comparing parents’ and caregivers’ perceptions of babycares, it was evident there was tension and disconnect between caregivers and parents due to conflicting views of a caregiver’s role in the babycares. These issues may present as barriers to resolving poor quality and establishing standards of babycares if not further explored or addressed.

Although parents and caregivers recognize that there is an unfair caregiver to baby ratio, and that babycare centers run for long hours daily, their perception of their roles in providing supports and care for the baby are different which may contribute to conflicts in relation to what caregivers should be trained to do.

Caregivers often cook food for the babies they care for but they felt that parents should be responsible for providing food to their children. In contrast, parents felt the caregiver ought to prepare and serve food. This presents an issue in terms of determining whether caregivers should be trained in appropriate food preparation and safety.

Caregivers stated they had some resources for these children but expected parents to bring toys and items for the baby. Overall, caregivers expressed the need for more resources including toys, mattresses for babies to sleep on, and other personal care items for babies. This conflict would present an issue on who’s responsibility is it to provide resources for babies and children to use.

One parent said:

*“I take her with packed food, but, if it got finished, the babycare prepares food, but, before giving the baby, they will ask you if there is a person at home who can bring her food*. ***”*** (*Parent, FG1*).

Conversely, a caregiver when asked about roles of a parent said:

*“Some parents do not clean the lunch boxes well so the food goes bad and you are forced to prepare fresh food*.*”* (Caregiver, FDG2).

Notably, some caregivers and parents felt the caregivers had the right to discipline the children, with some parents and caregivers permitting corporal punishment.

*“They should be told whatever they are doing is not right*. *They should be beaten*.*”* (*Parent, FG2*).

While one parent did not agree,

*“They should be corrected calmly*. *You can negotiate with them they do not have to be beaten*.*”* (*Parent, FG1*).

To implement the appropriate standards related to the space of babycares and training, parents and caregivers need to agree on what each of their role is in working and participating in babycares.

## Discussion

This study is one of the first of a series of studies aimed at understanding parents’, caregivers’, and community elders’ perspectives of babycares in the urban informal settlement of Mlolongo, Kenya. The results of the study were used to inform the development of community-engaged strategies to improve early childcare in this area. Babycares in Mlolongo were generally of poor quality, did not adhere to recommended standards (24), and lack consistency across centres within the same communities.

Babycares do address barriers families face in securing childcare given their economic and employment situation they face. Personal, financial, and socioeconomic factors play a role in the type of babycare centre parents choose and this is associated with the quality of babycares that develop (25). Parents, caregivers, and elders recognize the existing limitations, support the development of standards, and feel more formalized training of caregivers is required.

However, there is disagreement and conflict between parents and caregivers in relation to expectations of babycare centres and this difference in perception may make it difficult to improve quality and implement standards. These challenges also extend between parents’ perceptions and the perceptions of elders and caregivers. Caregivers and elders often cite the mother’s ability and time to parent as reasons for problems with babycares while parents cite the lack of training among caregivers and resources at babycares as a reason for sub-optimal standards. Caregivers felt some parents increased their workload by not preparing food well or picking up their children late.

The differences seen between parents, elders and caregivers is consistent with the literature. Service quality in children is often referred to as a subjective construct where the perception of quality involving the nature of childcare is based on the perceptions and backgrounds of those involved (26). In this case, caregivers perceive parents to contribute to their child in specific ways while parents expect caregivers to perform specific duties. To appropriately address the reported substandard of babycares, this conflict between parents and caregivers needs to be resolved by determining clear roles and responsibilities for each group working and participating in babycares. Also activities to promote a healthy caregiver and parent relationship are required to address any quality issues and the development of standards (25, 27).

In Mlolongo, there is great variability in the caregiver to baby ratio, ranging from eight to 20 babies per caregiver. Caregivers have reported stress in having to manage many babies at the same time and recommended additional personnel to improve the ratio. This continues to be an issue across other informal settlements and the inappropriate ratio is reportedly impacting early child development and quality of care. Current daycare and babycare centre standards recommend one caregiver for every 3-4 children (28).

Resources are inconsistent between babycares, while some may have beds and toys for babies, others require parents to bring these items. Both caregivers and parents agree that current babycares have limited resources and personnel, however, their perceptions of their roles differ. While parents have reported they feel caregivers are responsible for providing food for their children, caregivers expect parents to bring food and sleepwear. Both parents and caregivers recognize that personnel are limited but both groups feel that it is not their responsibility to provide resources or time to address this gap. A transparent understanding of caregivers’ and parents’ roles is required to ensure that a trustworthy, sustainable relationship is established between caregivers and parents in the community (25, 27).

Meeting standards in resources, cleanliness, and hygiene for babycares are important for healthy early child development. Babies attending babycares are at a higher risk of infectious diseases than those who do not attend babycares (29). The implementation of evidence-based practices into babycares would result in better hygiene practices and healthier food for these children. As such, practice standards can reduce the incidence of illness and improve developmental and nutritional outcomes for children at risk. Babycares and daycare centres are seen as important places for children at risk to make nutritional and developmental gains given the amount of time children spend in these environments (1). As such, standards for babycares should support optimal opportunities for children to have sufficient healthy meals and to have setting supportive of play. This is especially beneficial in urban informal settlements where families have limited resources (30).

In this study, elders, parents, and caregivers agreed on the following standards for babycares; (1) reduced caregiver-to-baby ratio, (2) sufficient resources and personnel to meet the demands of each babycare centre, (3) transparent agreement between parents’ and caregivers’ roles at babycares, and (4) appropriate training for caregivers. This level of agreement among key stakeholders provides the foundation for community development of standards and improvement in quality. The findings are consistent with a previous study looking at babycares in Nairobi, Kenya (14).

There were limitations to this study. The study used purposive sampling which may have introduced bias in the findings. Views of those unable to attend may have been missed. However, the views between those who did attend were consistent. Also, some parents were also caregivers working in babycares and this may have influenced their perceptions.

This was one of the first studies to explore the perceptions of key stakeholders - caregivers, parents, and elders - on the current quality of babycares in informal community settings. These settings are important given the existing and increasing number of children living in informal communities associated with urbanization. Investment in community-supported standard setting, quality improvement strategies and monitoring in relation to standards is supported by community stakeholders. Quality childcare resources invested in these communities can have significant positive outcomes for vulnerable children who are exposed to multiple risks that impact their health and development.

## Data availability statement

The raw data supporting the conclusions of this article will be made available by the authors, without undue reservation.

## Ethics statement

The studies involving human participants were reviewed and approved by the AKU Ethics Review Committee. The patients/participants provided their written informed consent to participate in this study.

## Author contributions

RM, MM, AY, VS, and RA contributed to the study design. RM, MM, and VS were responsible for the implementation of the study. TJ and RM shared equally the first draft of the paper. All authors contributed to core content and revisions and approved the final paper.

## Funding

The study was funded through a grant to the Aga Khan Foundation Canada from the Canadian Government under the Institutional Partnerships for Human Development (IPHD)/Partnership for Advancing Human Development in Africa and Asia (PAHDAA). VS was seconded to Aga Khan University for this study while enrolled and financially supported through the Johnson & Johnson Talent for Good Program.

## Conflict of interest

MM and RA are currently partners in the Health Associates GmbH, Germany, a consulting service at the time of publication. They were employed by AKU when the study was carried out. RA is currently Emeritus faculty at AKU. VS is employed by Johnson & Johnson and was on secondment to AKU during the implementation of this study.

The remaining authors declare that the research was conducted in the absence of any commercial or financial relationships that could be construed as a potential conflict of interest.

## Publisher’s note

All claims expressed in this article are solely those of the authors and do not necessarily represent those of their affiliated organizations, or those of the publisher, the editors and the reviewers. Any product that may be evaluated in this article, or claim that may be made by its manufacturer, is not guaranteed or endorsed by the publisher.
