# Supplementary material for: Informal settlements and the care of children 0–3 years of age: a qualitative study
Source: Front Public Health. 2023 Aug 23;11:1110578. doi: 10.3389/fpubh.2023.1110578 (PMC10482249; doi:10.3389/fpubh.2023.1110578)
Supplement: Supplementary file 1 [file Table_1.DOCX]

**Appendix A:**

**Improving Care for Young Children in Mlolongo**

Interview Guide

*Families*

We are working in conjunction with Aga Khan University Hospital. During the focus group we will ask you to provide information on the babycare centre your child attends.

First, please tell us about yourself, and your child/children.

Interview Guide:

1. Please tell us about your day to day routine. When you wake up in the morning what do you do? How do you get your child ready for babycare centres? How long does your child stay in the babycare centre?
2. How is your relationship with babycare staff?
3. What did you like about the babycare centres?
4. What would you like to see changed about the babycare centres?
5. What, if any, were the benefits to your child from being in the babycare centres?
6. What, if any, were your concerns about your child being in the babycare centres?

**Improving Care for Young Children in Mlolongo**

Interview Guide

*Babycare Staff*

We are working in conjunction with Aga Khan University Hospital. During the focus group we will ask you to provide information on your experience working in babycare centres.

We will largely discuss your experience with the babies/children that are brought to your babycare centres. about the babies who are brought to our baby cares.

Interview Guide:

1. How did you start working at a babycare centre?
2. How many babies/children do you take care of?
3. How do we prepare to receive these babies?
4. What resources do you use to care for the babies? Are they yours or do parents bring it with them?
5. How is our relationship with the mothers?
6. What experience do you have in dealing with the mothers and the babies?
7. What, if any, are the benefits of babycare centres?
8. What, if any, were your concerns about your babycare centres?
9. What would we want to be improved in our babycares centres?

**Improving Care for Young Children in Mlolongo**

Interview Guide

*Community Elders*

We are working in conjunction with Aga Khan University Hospital. During the focus group we will ask you to provide information on your knowledge of babycares in your community

Interview Guide:

1. As a leader are you aware of what happens at the baby care centres? What activities take place at the baby care centres?
2. What did you like about the babycares?
3. How safe are the babies in babycares?
4. What makes parents bring their babies/children to babycares?
5. What would you like to see changed about the babycares?
